# In vitro comparative evaluation of the flexural strength of acrylic denture bases reinforced with nano-PEEK and PEEK–zirconia composites

**DOI:** 10.1038/s41598-026-36102-3

**Published:** 2026-02-06

**Authors:** Sara Alrais, Ibrahim Alghoraibi, Alaa Salloum

**Affiliations:** 1 Department of Removable Prosthodontics, Faculty of Dental Medicine, Damascus University, Damascus, Syria; 2 Department of Physics, Faculty of Science, Damascus University, Damascus, Syria; 3 Department of Basic and Supporting Sciences, Faculty of Pharmacy, Arab International University, Ghabaghib, Dara, Syria

**Keywords:** PMMA, Zirconia, PEEK, Nanocomposite, Denture base and flexural strength, Materials science, Medical research, Nanoscience and technology

## Abstract

Poly(methyl methacrylate) (PMMA) is the most widely used denture base material but exhibits limited fracture resistance under functional loads. This study investigates the effect of incorporating zirconia (ZrO_2_) and polyether ether ketone (PEEK) nanoparticles on the structural and mechanical properties of PMMA. Thirty rectangular heat-cured PMMA specimens (65 × 10 × 2.5 mm) were divided into three groups: Group C (control): unmodified PMMA; Group P: PMMA with 5% PEEK nanoparticles; and Group ZP: PMMA with a hybrid of 2.5% ZrO_2_ and 2.5% PEEK nanoparticles. Nanoparticle morphology was analyzed by field emission scanning electron microscopy (FESEM), showing PEEK sizes between 26 and 100 nm and spherical ZrO_2_ with a mean diameter of 47 nm. Energy-dispersive X-ray spectrometry (EDXS) was performed on ZrO_2_ only. FTIR analysis confirmed interfacial bonding in the hybrid composite. Flexural strength was measured via a three-point bending test. The ZP group exhibited the highest flexural strength (mean: 139.78 MPa), significantly outperforming both P and C groups (*p* < 0.05), with no significant difference between P and C. The synergistic addition of ZrO_2_ and PEEK nanoparticles significantly improved the mechanical and structural behavior of PMMA, offering a promising strategy to enhance durability and clinical performance of denture base materials in prosthodontics.

**Supplementary Information:**

The online version contains supplementary material available at 10.1038/s41598-026-36102-3.

## Introduction

Although dental implants have become popular everywhere for the treatment of edentulous patients, complete dentures remain a significant form of treatment on medical and economic reasons^1^. Acrylic resins have remained the preferred material for complete denture bases due to their favorable properties^2^. Fractures of the denture are usually seen and have been attributed to denture base deformation during function, leading to flexural fatigue failure^3^. Midline fractures are extremely prevalent in complete upper denture patients and usually occur due to chronic flexing and bending^4^. To provide maximal clinical function, denture base resins should successfully resist crack progression and impact loads. Therefore, enhancement of the mechanical properties of PMMA-based materials is required to fabricate dentures with improved fracture resistance^5^. Many methods have been applied to improve physical properties of denture base materials like chemical modification of PMMA, utilization of substitute materials, inclusion of fiber reinforcements, and incorporation of macro- and nano-fillers^6,7^. Nanomaterials possess excellent characteristics compared to bulk material^8,9^. Incorporation of nanomaterials into a polymer matrix was found to improve optical and mechanical performances of nanocomposites^9^. Numerous investigations have been conducted on enhancing denture base materials by incorporating different forms and concentrations of nanoparticles such as Titanium Dioxide (TiO_2_)^10–12^, Silver (Ag)^12,13^, Silicon Dioxide (SiO_2_)^14,15^, Aluminum Oxide (Al_2_O_3_)^16,17^, Copper Oxide (CuO)^18^, Zinc oxide (ZnO)^19^ Strontium titanate^20^, and Zirconium dioxide (ZrO_2_)^21^, Polyether ether ketone (PEEK) is an amorphous thermoplastic polymer with a semi-crystalline structure, possessing superior thermal stability, excellent mechanical properties, and high wear resistance. PEEK is resistant to hydrolysis, non-toxic, and highly biocompatible^22,23^. Especially, its mechanical properties remain unaffected after being exposed to various sterilization procedures, including steam sterilization, gamma irradiation, and ethylene oxide exposure^24,25^. ZrO_2_ is an inorganic filler and has been of great interest for polymer nanocomposites’ property improvement^26^. The mechanical properties of zirconia are superior, with flexural strength of 900–1200 MPa, a hardness of 1200 HV, and fracture toughness of 9–10 MPa·m^1/2^. Zirconia is also corrosion resistant and biocompatible and hence a good candidate for reinforcement of denture base polymers^27^. Nanoparticles exhibit high surface energy and polarity, and the uniform distribution of hydrophilic surfaces throughout the polymer can be challenging, leading to material aggregation and phase separation^28^. The nanoparticle surface must be modified with bonding agents such as silane^28^, which can create a strong interfacial bond with the resin material^29^. Using the silane bonding agent to treat the surface of zirconia nanoparticles reduces the agglomeration of nanoparticles and improves the distribution within the polymer material^30,31^. Given the scarcity of studies on this topic, it was necessary to understand the effect of adding polyether ether ketones and polyether ether ketones and zirconia together in denture base resins.

The aim of the present investigation was to contrast the effect of adding 5% PEEK nanoparticles or a mixture of 2.5% zirconia nanoparticles and 2.5% PEEK nanoparticles on the denture base acrylic resin’s flexural strength. The null hypothesis was that adding silane-treated PEEK nanoparticles, or a mixture of silane-treated zirconia and PEEK nanoparticles, would not increase the flexural resistance of the denture base acrylic resin.

## Materials and methods

### Preparation of specimens

The sample size was calculated using G. Power 3.1.9 software (G*Power 3.1.9, Heinrich Heine Universität Düsseldorf, Düsseldorf, Germany). A sample size was obtained based on the study by Mustafa et al.^32^. (Effect size f = 1.707, α err prob = 0.05, power (1 − β err prob) = 0.95, number of groups = 3). The result was 9, and to avoid the borderline value of 27, 30 was used, based on the Zeidan et al.^33^.

The 5% weight ratio was adopted based on the study by^33–35^, who found that this ratio gave the highest flexural strength. To standardize the added acrylic ratio, it was adopted in both groups. In the hybrid group, the total ratio of the two added materials was 5%.

Thirty rectangular specimens (65 × 10 × 2.5 mm) were obtained from heat-cured acrylic resin (Vertex Heat-Cured Acrylic, Vertex Dental, Zeist, The Netherlands). The specimens were sectioned into three groups in a balanced manner according to the kind and concentration of nanoparticles added to the resin:

- Group C: Control group (0 wt% nanoparticles).
- Group P: 5 wt% nano-PEEK.
- Group ZP: 2.5 wt% nano-ZrO_2_ + 2.5 wt% nano-PEEK.

Four rectangular molds (65 × 10 × 2.5 mm) were fabricated using addition-type standard duplicating silicone (Mega Dental Materials, Slovakia and Czech Republic) according to ISO 1567 specifications. The bottom half of a copper dental flask was filled with a dental stone mixture. A glass plate, matching the dimensions of the flask, was placed on top. The silicone molds were affixed to the glass plate using an appropriate adhesive to prevent displacement during the investment procedure. The upper section of the flask was filled with an improved dental stone mixture (Type IV dental stone, Shera Hard Rock, Shera, Germany), covered, and pressed using a hydraulic press. After the dental stone had set, the flask was opened and the silicone molds were carefully removed. Finally, the stone surface was treated with a silicate coating.

### Size of nano-ZrO_2_ and nano-PEEK particles

**Fig. 1:**
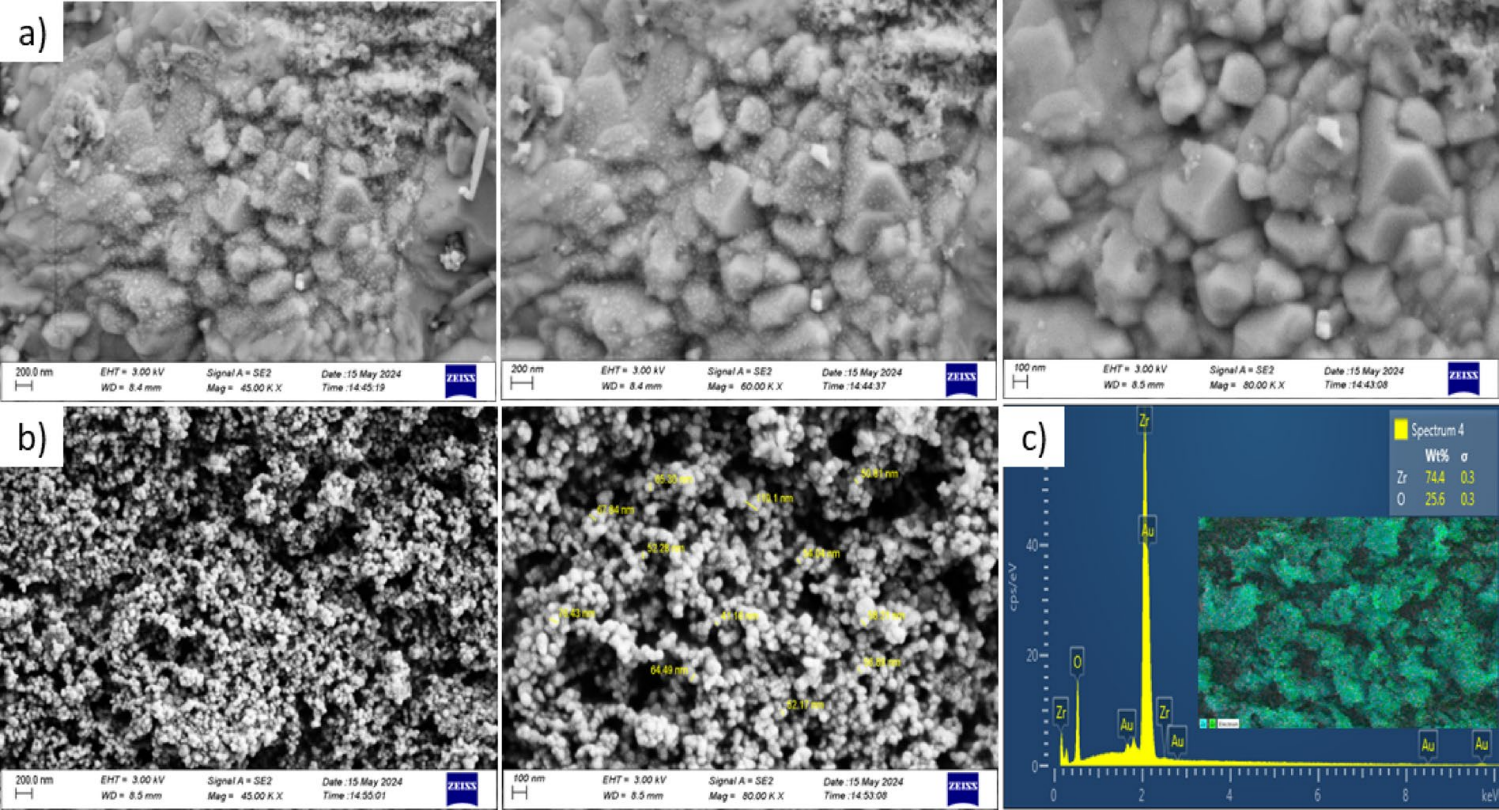
FESEM images of (**a**) nano-PEEK and (**b**) nano-ZrO_2_ particles, and (**c**) EDX spectra and elemental mapping of nano-ZrO_2_.

The particle size and morphology of the nano-ZrO_2_ and nano-PEEK powders were examined with FE-SEM at different magnifications. The Nano-ZrO_2_ particles were mostly spherical and very uniform in shape and distribution with an average particle size of about 47 nm (Fig. 1b). FESEM micrographs on two magnifications (45,000 × and 80,000 ×) reflected this uniformity and lack of any extreme agglomeration, indicating good nanoparticle dispersion. Contrarily, nano-PEEK particles were characterized by a wide distribution in size from 26 to 100 nm and exhibited a distinctly cubic-like morphology. FESEM micrographs at 45,000 × , 60,000 × , and 80,000 × magnifications showed distinct (Fig. 1a), angular boundaries of particles and faceted surfaces characteristic of crystalline structures, which confirmed that PEEK nanoparticles are of well-defined, ordered morphology to further elucidate the elemental nature of the zirconia nanoparticles, EDX was performed (See Fig. 1c). The data indicated a high-purity composition of 74.6 wt% of zirconium (Zr) and 25.4 wt% of oxygen (O) with no detectable impurities. A small gold (Au) peak was also observed, which was caused by thin gold coating placed on the imaging sample surface to ensure conductivity. EDX elemental mapping further confirmed the homogenous distribution of Zr and O in the sample region, thus the structural homogeneity of the formed nano-ZrO_2_ powder. Additional FESEM micrographs of nano-zirconium oxide and nano-PEEK at various magnifications are provided in Supplementary Figs. S2 and S3, respectively, while the elemental mapping analysis of nano-ZrO_2_ is presented in Supplementary Fig. S1, including detailed elemental distribution maps.

**Fig. 2:**
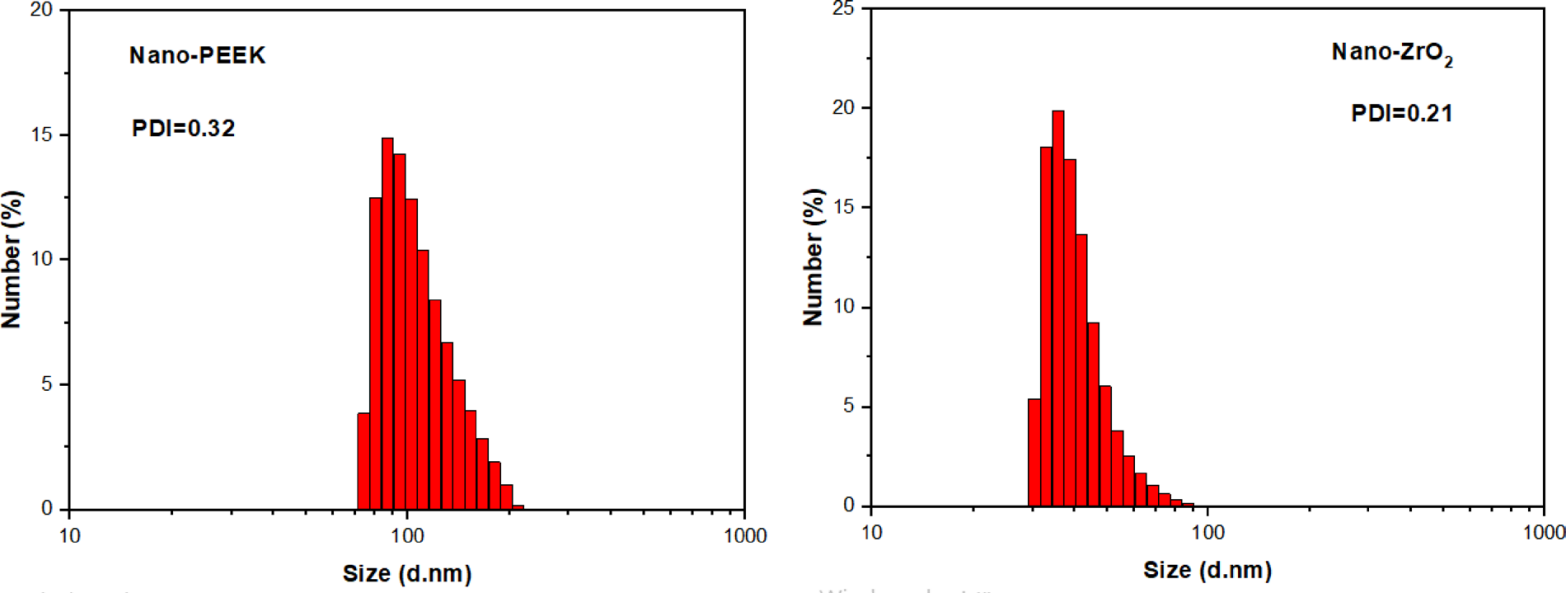
Size distribution of nano-ZrO_2_ and nano-PEEK powders.

Complementing the morphological results from FESEM, DLS analysis was carried out to evaluate particle-size distribution and dispersion quality of both nano-ZrO_2_ and nano-PEEK powders (See Fig. 2). The DLS analysis indicated that nano-ZrO_2_ has a narrow size distribution with most of the particles falling between 30 and 90 nm with a PDI value as low as 0.21, hence representing very uniform and monodispersed nanopowder. This is in good agreement with the FESEM images showing spherical and well-distributed morphology.

By contrast, the nano-PEEK particles exhibited a wider hydrodynamic size distribution, ranging approximately between 70 and 200 nm, with a PDI of 0.32. Whereas this is broader than that of zirconia, this value remains acceptable for polymer-derived nanomaterials. The DLS profile agreed with the observations from FESEM that showed cubic-like faceted morphology with a wider size distribution range of 26–100 nm. Overall, these FESEM and DLS analyses confirm that both nanopowders have suitable nano-scale dimensions and good dispersion characteristics for effective incorporation into the acrylic resin matrix.

### Preparation of PMMA/PEEK and PMMA/PEEK, ZrO_2_ nanocomposites

The specified quantities of PMMA, nano-ZrO_2_, and nano-PEEK for blending were precisely determined based on the aforementioned ratios using electronic scales accurate 0.0001 g. The zirconium dioxide and PEEK nanoparticles' surfaces were coated with silane, subsequently dried, and combined with acrylic resin powder.

### Processing of specimens

The fabrication of specimens was conducted according to standardized procedures to ensure consistency and reliability. The heat-polymerized acrylic resin (Vertex Heat Cured Acrylic, Vertex Dental, Zeist, The Netherlands) was prepared by mixing the powder and liquid components in the manufacturer's recommended ratio of 2.3 g powder (polymer) to 0.95 g liquid (monomer). The mixing process was carried out manually until a dough-like stage was achieved. The dough was then packed into the pre-fabricated silicone molds (65 × 10 × 2.5 mm), following the ISO 1567 specifications. After packing, the molds were sealed and subjected to hydraulic pressing to ensure uniform compaction and removal of air voids. Subsequently, the flasks were securely clamped and placed in a HANO curing unit at 212°F (100 °C) for 20 min, in accordance with the manufacturer's curing protocol. Upon completion of the polymerization cycle, the flasks were allowed to cool naturally at room temperature for 30 min to relieve internal stresses, followed by immersion under running tap water for 15 min to complete the cooling process.

**Fig. 3:**
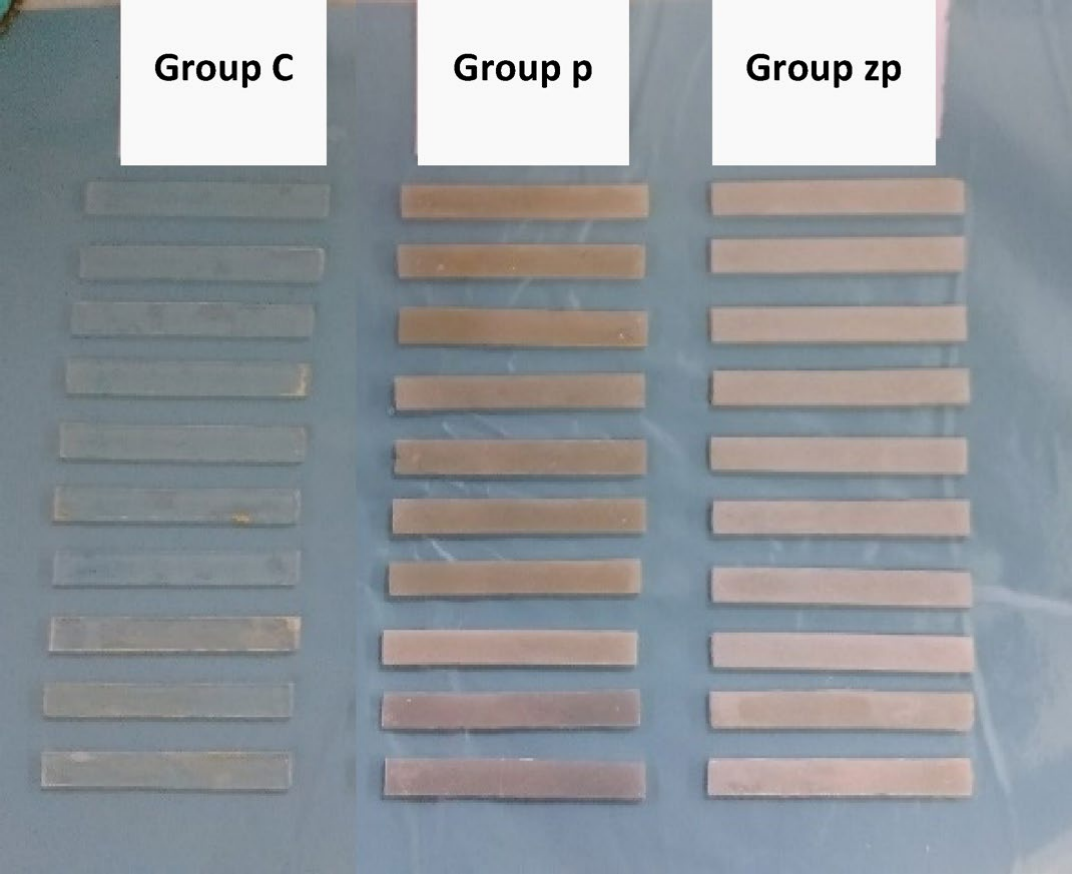
Specimens of the three groups after finishing.

Afterward, the flasks were carefully opened, and the cured specimens were retrieved. Specimens were then finished using silicon carbide abrasive papers of progressively finer grits to achieve smooth surfaces, minimizing surface defects that could affect mechanical testing. All specimens were visually inspected to ensure the absence of visible defects such as air bubbles, porosities, or surface irregularities before immersed in artificial saliva and mechanical testing (Fig. 3).

### Preparation of artificial saliva and sample incubation

Artificial saliva was prepared according to the formulation by Imai and Takashima^36^ as detailed in Table 1. The solution contains inorganic salts (K_2_SO_4_, KCl, Ca_3_(PO_4_)_2_, KH_2_PO_4_, Na_3_PO_4_) and albumin dissolved in distilled water to simulate the chemical environment of natural saliva. Specimen were immersed in the artificial saliva and incubated under controlled conditions for 1 month to mimic the oral environment. This procedure enables evaluation of the samples’ physicochemical stability and surface properties in conditions resembling the oral cavity. The samples were immersed in artificial saliva and placed in the incubation device for 1 month.

**Table 1:** Composition of the artificial saliva used in the study according to Imai and Takashima^36^ showing the precise quantities of each component dissolved in 100 g of distilled water.

Material	Quantity (g)
K_2_SO_4_	0.09
KCl	0.24
Ca_3_PO_4_	0.06
KH_2_PO_4_	0.14
Na_3_PO_4_	0.08
Albumin	0.5
Distilled water	100

### Three-point bending test

**Fig. 4:**
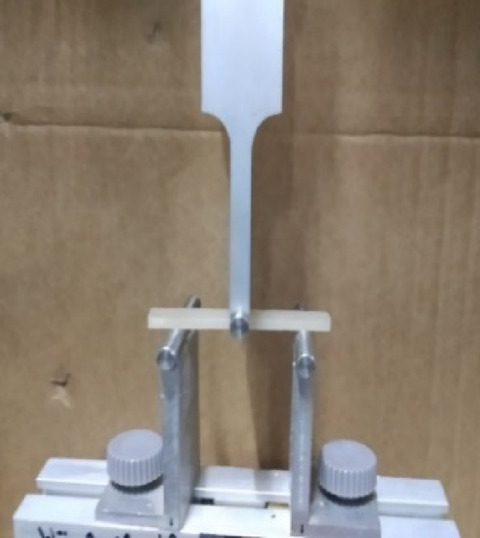
Three-point bending test.

The flexural strength of the specimens was evaluated using a three-point bending test performed on a Universal Testing Machine (DY-34 ADAMEL LHOMARGY, FRANCE) (Fig. 4). The test was conducted at a crosshead speed of 10 mm/min, with the load applied centrally until fracture occurred. The maximum load (P) recorded at the point of fracture for each specimen was used to calculate the flexural strength (S) according to the following formula: *S* = *3PL/2bd*, Where S = Flexural strength (N/mm), P = Load at fracture (N), L = Span length (distance between the two supporting jigs) (50 mm), b = Width of the specimen (mm), d = Thickness of the specimen (mm). All specimens were carefully aligned during testing to ensure accurate and reproducible results.

## Result

### Fourier-transform infrared spectroscopy (FTIR)

**Fig. 5:**
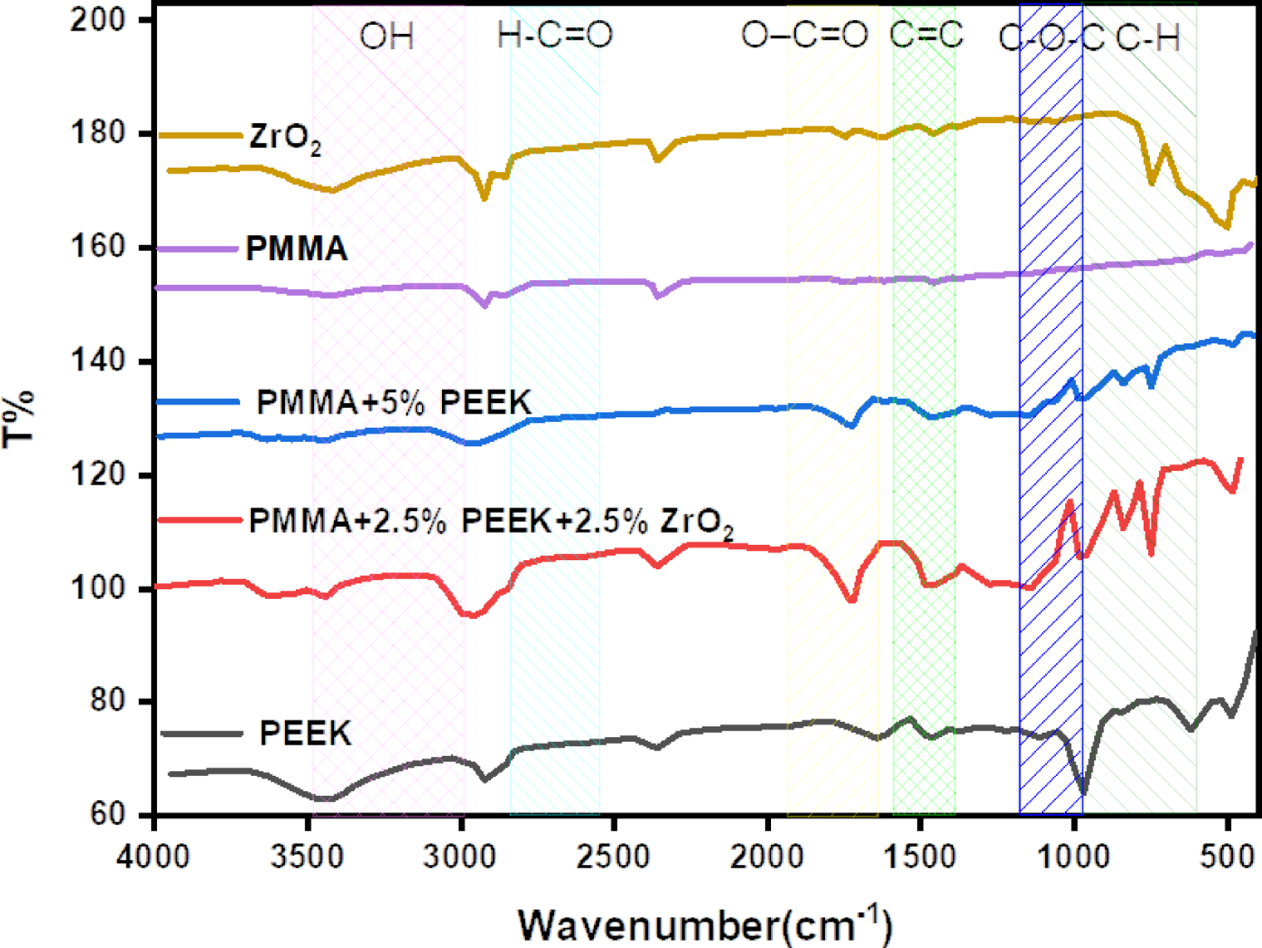
FTIR spectra of ZrO_2_, PMMA, PEEK, PMMA + 5% ZrO_2_, PMMA + 5% PEEK, and PMMA + 2.5% PEEK + 2.5% ZrO_2_.

FTIR spectra of individual and blended systems ZrO_2_, PMMA, PEEK, PMMA + 5% PEEK, and PMMA + 2.5% PEEK + 2.5% ZrO_2_ are shown in Fig. 5. The spectra exhibit characteristic vibration features that provide information on functional groups and interactions between constituents in each composition. There is a broad band of absorption in the ZrO_2_ spectrum at 3400–3200 cm^−1^ that is due to stretching vibrations of hydroxyl (O–H) groups on the surface adsorbed with moisture or terminal hydroxyl groups on the oxide surface. In the usual fashion, characteristic Zr–O vibrations are observed as strong, broad bands in the fingerprint region between 700 and 500 cm^−1^. The PMMA spectrum displays its characteristic ester carbonyl stretching vibration at ~ 1730–1740 cm^−1^, as well as intense C–O–C stretching vibrations between 1200 and 1000 cm^−1^. Aliphatic C–H stretching vibrations are present between 2950 and 2850 cm^−1^. These bands will be used as reference values in identifying the interactions within the blend systems. Broadening and red-shift are mild indicators of strong interaction of the polar carbonyl unit with the Zr^4+^ sites on the nanoparticle surface through dipole–ion or hydrogen-bonding mechanisms. Furthermore, the widening of the C–O–C stretching band is a sign of structural rearrangement or partial coordination between the ester oxygen and the zirconia surface.

For the PMMA + 5% PEEK composite, new absorptions at 1650–1600 cm^−1^ and 1500 cm^−1^ are due to the C = O stretching mode of ketone groups and aromatic C=C stretching vibration of the PEEK backbone. These bands are absent in pure PMMA, confirming the existence of the aromatic polyether ether ketone backbone. Increased intensity in the C–O–C region also confirms the contribution of PEEK's ether linkages, confirming a successful molecular blend.

The most complex spectrum is the ternary system (PMMA + 2.5% PEEK + 2.5% ZrO_2_), where a combination of features of all three materials is seen. The ester carbonyl band widens significantly and shifts red slightly, as would be anticipated for dual interaction—hydrogen bonding or dipole–dipole interactions with both PEEK and ZrO_2_. The C=C and C=O bands of PEEK remain visible but overlapped to some extent, while the C–O–C region is clearly broader and less resolved. This complex pattern is characteristic of multi-site interactioincluding: physical entanglement between PMMA and PEEK chains, possible coordination of Zr^4+^ ions with ether or carbonyl functional groups, and hydrogen bonding between surface hydroxyl groups of ZrO_2_ and the carbonyl or ether oxygen atoms. In the hybrid PMMA/PEEK/ZrO_2_ composite, the characteristic Zr–O band typically observed at 500–750 cm^−1^ in pure zirconia was not clearly detected. This is attributed to the low ZrO_2_ loading (2.5 wt%) and the strong overlapping absorption bands of PMMA and PEEK in the same region, which attenuate and mask the weak Zr–O vibration signal.

The PEEK spectrum exhibits sharply resolved characteristic absorptions of ketonic and aromatic groups at approximately 1650 cm^−1^ and 1500 cm^−1^, respectively, along with intense stretching vibrations of ether linkages in the range of 1200–1000 cm^−1^. These serve as reference points for comparison with the hybrid formulations. Overall, FTIR results give evidence that the incorporation of ZrO_2_ and PEEK in the PMMA matrix yields unmistakable structure changes, and these may be evidenced through band shifts and broadenings of major vibrational bands. These are not additive but evidence real molecular-level interactions and hybrid network formation. The ternary composite exhibits increased spectral complexity, which indicates synergistic interfacial interactions that can enhance the mechanical strength, thermal stability, and barrier property of the material. Such molecular-level evidence is key to designing advanced polymer nanocomposites specifically for acrylic denture base strengthening and extending their durability and wear resistance.

The FTIR spectra of the reinforced groups demonstrated a slight but consistent shift in the PMMA carbonyl stretching band (C=O), in the range of 3–6 cm^−1^, indicating the presence of secondary interactions most likely weak hydrogen bonding—between the carbonyl groups of the polymer matrix and the nanoparticle surfaces. In addition, noticeable broadening within the C–O–C absorption region (1140–1180 cm^−1^) supports the occurrence of improved wetting and more effective dispersion of the nanofillers throughout the matrix. These spectroscopic modifications corroborate the proposed reinforcing mechanism, whereby enhanced interfacial compatibility promotes better load transfer, restricts crack initiation, and increases the resistance of the composite to crack propagation, ultimately contributing to the observed improvement in flexural strength.

The One-Sample Kolmogorov–Smirnov test was used to determine the data distribution pattern (Table 2).

**Table 2:** Results of the one-sample Kolmogorov–Smirnov test for determining the data distribution pattern.

Group		PMMA	PEEK	ZP
Number		10	10	10
Natural parameters	Average	124.464	113.67	142.98
	Standard deviation	9.588	7.895	18.490
The most extreme differences	Divorced	0.130	0.184	0.126
	Positivity	0.122	0.184	0.126
	Negativity	− 0.130	− 0.161	− 0.115
Kolmogorov–Smirnov Z		0.410	0.582	0.398
Probability value		0.996	0.887	0.997

We find from Table 2 that all groups have given a probability value exactly greater than 0.05, and therefore all groups have normally distributed data.

**Fig. 6:**
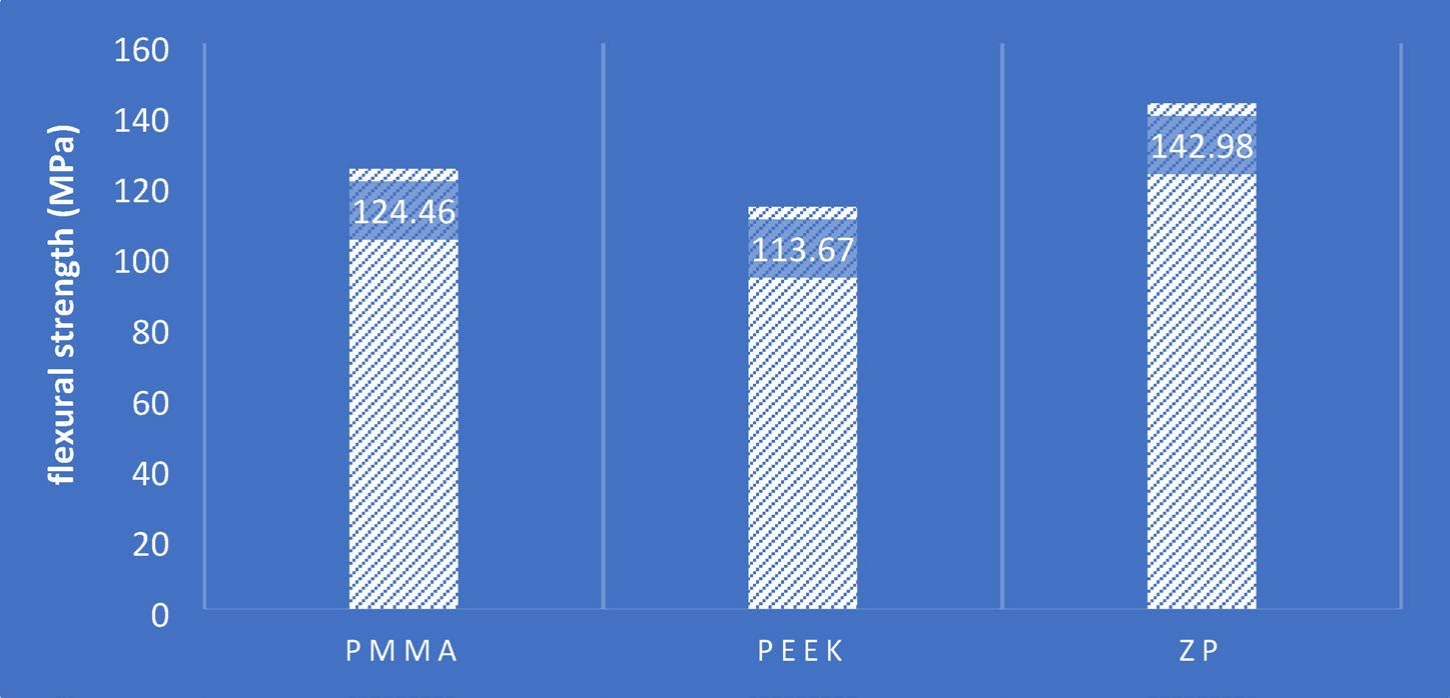
Bar chart showing the mean resistance to bending forces in the study groups PMMA, PMMA with 5% PEEK and PMMA with 2.5% PEEK + 2.5% ZrO_2_.

The flexural strength values of the tested specimens, presented in Table 3 and Fig. 6, demonstrate statistically and clinically significant differences among the groups. The hybrid-reinforced group with ZrO_2_ and PEEK nanoparticles (ZP) had the best mean flexural strength (142.98 MPa), which was superior to that of the pure PMMA group (124.86 MPa) and the pure PEEK group (113.67 MPa). The ZP group improvement was statistically significant (*p* < 0.05), as confirmed by one-way ANOVA and Tukey’s post hoc test. No significant difference was found between PMMA and PEEK groups.

**Table 3:** Descriptive data of the flexural strength recorded in each study group.

Group	Number	Highest value	Minimum value	Arithmetic mean	Standard deviation
PMMA	10	142.08	107.16	124.46	9.588
ZP	10	171.48	117.24	142.98	18.490
PEEK	10	130.92	105.84	113.67	7.895

Post-hoc analysis using Tukey's HSD test, presented in Table 4, revealed statistically significant differences between some group pairs for flexural strength. The ZP group exhibited significantly higher flexural strength than the PEEK group (*p* < 0.001) and the PMMA group (*p* = 0.011). No statistically significant difference was observed between the PMMA and the PEEK group (*p* = 0.236).

**Table 4:** Turkey’s Post-hoc comparisons between each of the two groups to find the significant differences in the averages of flexural strength between the study groups.

Group I	Group J	Average difference I–J	Standard error	Probability value
PMMA	PEEK	10.792^a^	5.605	0.236
	ZP	− 18.516^a^	5.605	0.011*
PEEK	ZP	− 29.308^a^	5.605	0.001*

^a^Tukey’s Post-hoc.

*Statistically significant.

**Fig. 7:**
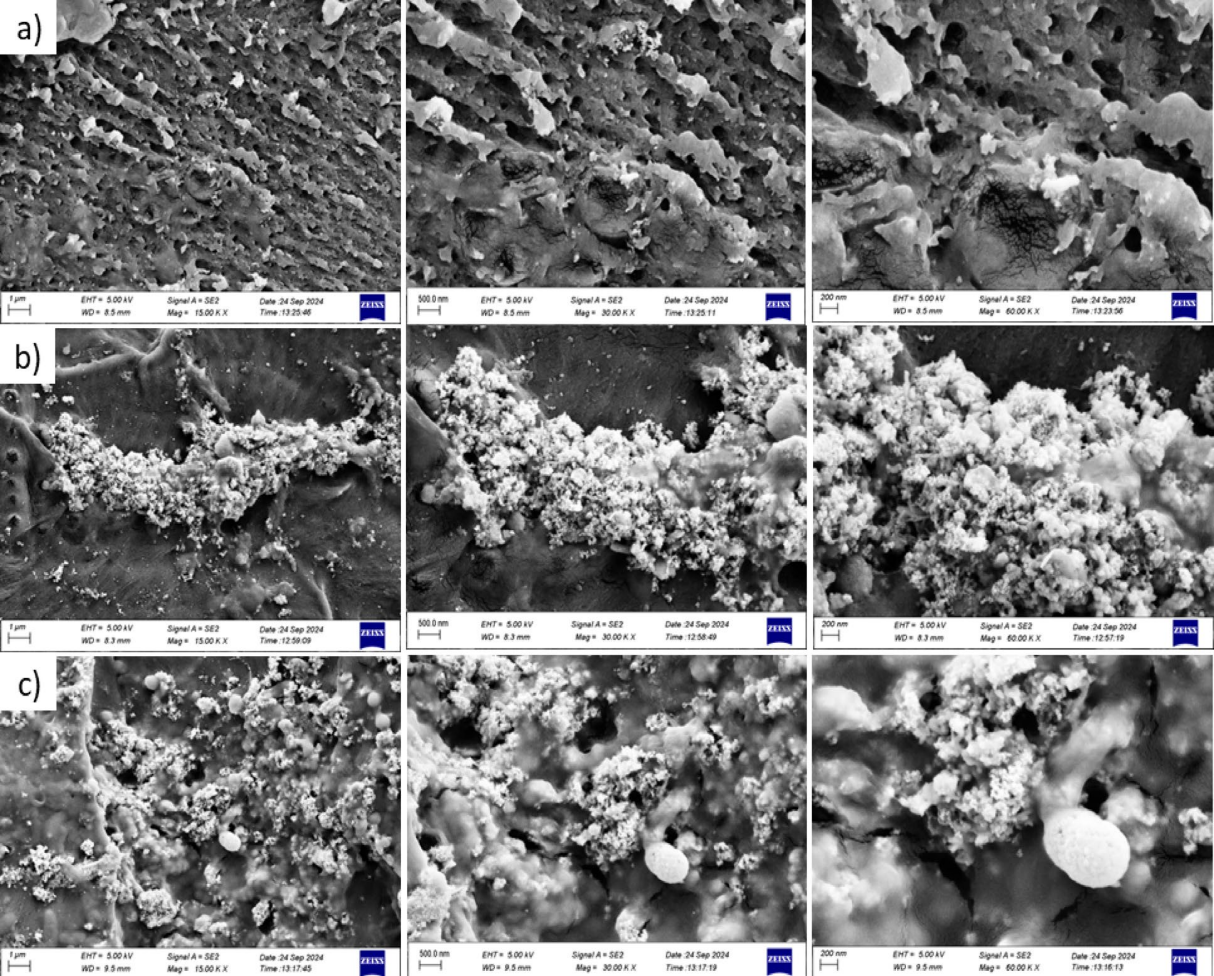
A cross-sectional FESEM images of the fracture surface of (**a**) PMMA, (**b**) PMMA with 5% PEEK and (**c**) PMMA with 2.5% PEEK + 2.5% ZrO.

The FESEM micrographs at different magnifications (15,000 × , 30,000 × , and 60,000 ×) in Fig. 7 display the cross-sectional morphology of the fracture surfaces of PMMA-based denture base specimens. In the control group (PMMA with no additives), the fracture surface showed a porous appearance with many visible voids, indicating weak internal cohesion and the inherently brittle nature of unmodified PMMA. In the 5% PEEK nanoparticle-reinforced group, evident agglomeration of the PEEK particles was noted, with poor and nonuniform dispersion in the polymer matrix.

These agglomerates served as localized stress concentration areas, forming structural weak points that explained the failure to improve the flexural strength over the control group. By contrast, the specimen reinforced with a hybrid of 2.5% zirconia and 2.5% PEEK nanoparticles presented a distinctly different morphology. The FESEM cross-section manifested the homogenous dispersion of nanoparticles, with the majority of internal voids eliminated and the matrix more compact and integrated. Such uniform dispersion facilitated improved stress transfer and interfacial bonding strength, confirming the hypothesis of a synergistic reinforcing effect. These results attest that the dual reinforcement approach using zirconia and PEEK nanoparticles accomplishes greater structural cohesion and improves the flexural performance and fracture resistance of PMMA composites significantly.

## Discussion

The present study aimed to evaluate the effect of incorporating nano-PEEK and a hybrid material consisting of nano-zirconia and nano-PEEK, treated with silane, on the flexural strength of acrylic denture bases. High flexural strength is crucial for denture bases, as unbalanced forces can lead to deformation of the base, which relies on the alveolar bone for support, ultimately causing irregular resorption of the alveolar bone. Therefore, denture bases must withstand deformation and fatigue under repeated loading^37^. The addition of zirconium oxide nanoparticles was adopted in this study because it has wide use in various fields of dentistry, in addition to being a non-toxic material that does not affect the oral mucosa and can be inserted into thermal acrylic resin bonds without any problems, as it is a material in the form of nanoparticles. This leads to homogeneity of the acrylic mixture and prevents the formation of gaps within it, and it can withstand the thermal changes that the acrylic resin is exposed to during the curing cycle^38^.

PEEK is a chemically inert compound, insoluble at room temperature in all conventional solvents except 98% sulfuric acid^39^. PEEK cages are commonly used due to their superior flexibility, radiolucency, and MRI compatibility^40,41^. They do not cause cytotoxicity and elicit minimal inflammatory responses. Importantly, PEEK cages have high abrasion resistance and elasticity similar to that of natural bone, which may enhance load-sharing and stress distribution^42^. Additionally, PEEK dental implants have shown less stress protection compared to titanium implants due to the close compatibility between the mechanical properties of PEEK and bone. PEEK is a promising material for both removable and fixed prostheses^43^. Temporary crowns designed using computer-aided design (CAD/CAM) and made of PEEK material showed better marginal fit and good fracture resistance^44^. The FPD framework used with PEEK material provided very high patient comfort and acceptance^45^. Although PEEK is already used as a leading material in spine surgery, orthopedics, and sports medicine, its use in dentistry has yet to gain traction. This may be due to the limited number of long-term clinical studies available on the use of PEEK in clinical dentistry. Therefore, further research is needed on PEEK polymers as alternatives to the long-used metals^46^. In light of the above, it was chosen in our study as an acrylic additive.

Heat curing acrylic resin was used as it is the most common and widely used material in making denture bases, and the traditional curing method is still the most widely used and common method because it is fast, cheap, and does not require the availability of special, complex, and expensive tools^47^.

The importance of this research lies in the incorporation of materials with exceptional properties, such as zirconia and PEEK, in nano-sized form into the acrylic resin of denture bases, aiming to enhance the mechanical properties. The null hypothesis of the study was partially rejected. The results revealed no statistically significant difference in flexural strength between the group reinforced with nano-PEEK and the control group. However, significant differences were found between the control group and the ZP hybrid group, with the ZP group exhibiting significantly higher flexural strength. Additionally, significant differences were observed between the PEEK-only group and the ZP hybrid group, with the latter demonstrating superior flexural strength.

The superior performance of the ZP hybrid group can be attributed to the homogeneous distribution of nanoparticles, as evidenced by field emission scanning electron microscopy (FESEM) imaging. In contrast, agglomeration of nanoparticles was observed in the nano-PEEK group, as demonstrated to increased flexural strength in the hybrid group compared to the control group may also be explained by the reduction in porosity and filling of voids within the acrylic matrix, as observed in FESEM images of the fracture surfaces. Conversely, the agglomeration presents in the nano-PEEK group created stress concentration points, leading to irregular stress distribution and a consequent decrease in flexural strength. These findings show that there is a synergistic reinforcing effect of ZrO_2_ and PEEK nanoparticles in the PMMA matrix. The rigid inorganic ZrO_2_ particles can act as fillers to improve crack resistance and redistribute applied stress more uniformly, and PEEK supplies toughness along with interfacial bonding through its semi-crystalline aromatic polymer character. This two-step reinforcement mechanism results in a more efficient stress transfer and improved structural integrity of the composite material. The larger standard deviation of the ZP group may be caused by variations of the nanoparticle dispersion or local agglomeration, but the overall trend confirms a significant improvement of mechanical behavior.

These mechanical findings are in agreement with FTIR results that revealed interactions at a molecular level between PMMA, PEEK, and ZrO_2_, indicating compatibility and the possibility of physical or chemical bonding at the interface. Thus, the addition of ZrO_2_ and PEEK nanoparticles into PMMA is a promising strategy to improve the flexural strength and durability of acrylic denture base materials. These findings validate the synergistic reinforcement effect of having a combination of ZrO_2_ and PEEK nanoparticles in the PMMA matrix to attain a better mechanical performance than that of each filler on its own.

This study utilized silane treatment to achieve a homogeneous dispersion of nanoparticles. To the best of our knowledge, it represents the first attempt to incorporate silane-treated nano-PEEK into acrylic denture bases. Homogeneous nanoparticle distribution was achieved only in the ZP hybrid group, whereas it was not attained in the nano-PEEK group. Our findings are consistent with those of Barapatre et al.^48^ who reported that a hybrid nanoparticle reinforcement resulted in higher flexural strength compared to both the control group and the PEEK-only group. However, in contrast to Barapatre et al.^48^ our study found no significant enhancement in the flexural strength of the PEEK-only group compared to the control. This discrepancy may be attributed to differences in the nanoparticle concentration (3% used in their study) or the method of incorporation.

Similarly, our results differed from those reported by Mustafa^32^ who observed increased flexural strength in the PEEK-only group relative to the control. Such variation could be explained by differences in nanoparticle size, the method of nanoparticle incorporation, or the concentrations used (1%, 2%, and 3%).

Based on these results, future research should investigate:

- Hybrid nano-material contents of variables to determine the best reinforcement ratio.
- Long-term durability of nano-reinforcement on impact strength, fatigue life, and water absorption.
- Silane-treated nano-PEEK and nano-zirconia's oral cavity acceptability and cytotoxicity.
- Clinical trials to confirm the results established in the lab and evaluate the performance of nano-reinforced denture bases under service conditions.

## Conclusion

In the context of the current research, one is thus able to deduce that the addition of a hybrid nano-material made of silane-treated zirconia and PEEK nanoparticles greatly improved the flexural strength of acrylic denture bases over both the control and the PEEK-reinforced single groups. The utilization of nano-PEEK alone, however, did not improve the flexural strength of acrylic denture bases appreciably over the control group. The better performance of the ZP hybrid group is due to the uniform dispersion of nanoparticles and the lack of internal voids, which was ensured by field emission scanning electron microscopy (FESEM) experiments. The lack of nanoparticle agglomeration in the hybrid group enabled more uniform stress distribution and better mechanical performance.

## Supplementary Information

Below is the link to the electronic supplementary material.

Supplementary Material 1

## Data Availability

The data used to support the findings of this study are available from the corresponding author upon request.
