# Supplementary material for: In vitro comparative evaluation of the flexural strength of acrylic denture bases reinforced with nano-PEEK and PEEK–zirconia composites
Source: Sci Rep. 2026 Feb 6;16:7601. doi: 10.1038/s41598-026-36102-3 (PMC12936162; doi:10.1038/s41598-026-36102-3)
Supplement: Supplementary file 1 — Supplementary Material 1 [file 41598_2026_36102_MOESM1_ESM.docx]

**Flexural strength of acrylic denture bases reinforced with nano-peek and peek-zirconia composites: In vitro comparative study**

**Authors:** Sara Alrais^1^, Ibrahim Alghoraibi*****^2,3^ and Alaa Salloum^1^

**Authors address:**

1. Department of Removable Prosthodontics, Faculty of Dental Medicine, Damascus University, Damascus, Syria

2. Department of Physics, Faculty of Science, Damascus University, Damascus, Syria

3. Department of basic and supporting sciences, Faculty of Pharmacy, Arab International University, Syria

**Authors Emails:**

Sara alrais: saraalrais1996 @ gmail.com

Alaa salloum: dr.alaa.salloum @gmail.com

Ibrahim.alghoraibi: Ibrahim.alghoraibi@gmail.com

**Academic Dagree:**

Sara alrais; DDS-MSc student, Department of Removable Prosthodontics.

Alaa salloum**:** Professor Dr, Department of Removable Prosthodontics.

Ibrahim alghoraibi**:** Associate Professor Department of Physics**.**

**ORCID** **Number:**

Ibrahim alghoraibi: 0000-0002-7609-9137

Sara alrais; 0009-0001-9469-671X

***Corresponding Author**: Ibrahim alghoraibi**,** Associate Professor Department of Physics, Damascus University, Damascus, Syria

Phone number: 0934413442

[Ibrahim.alghoraibi@gmail.com](mailto:Ibrahim.alghoraibi@gmail.com)

**SI Figure**

**Fig. S1:** Elemental mapping of nano-ZrO₂.

**Fig. S2:** FESEM images of nano-ZrO₂ particles with different maginfication 5kx, 15kx, 30kx and 60kx.

**Fig. S3:** Fig. S3. FESEM images of nano-polyether ether ketone (nano-PEEK) particles recorded at different magnifications (5k×, 15k×, 30k×, and 60k×). The particles exhibit a cubic-like morphology, along with the presence of smaller nanoparticles deposited on the surfaces of the larger cubic particles.


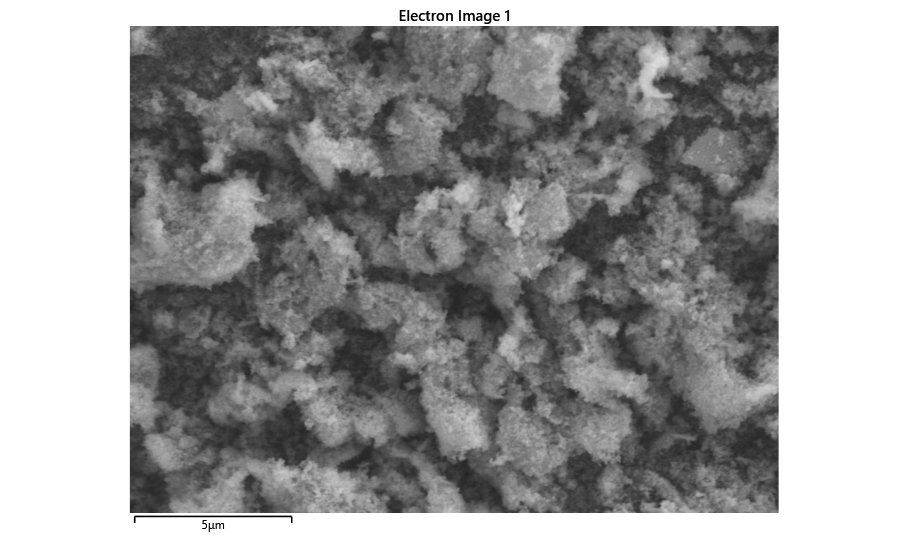


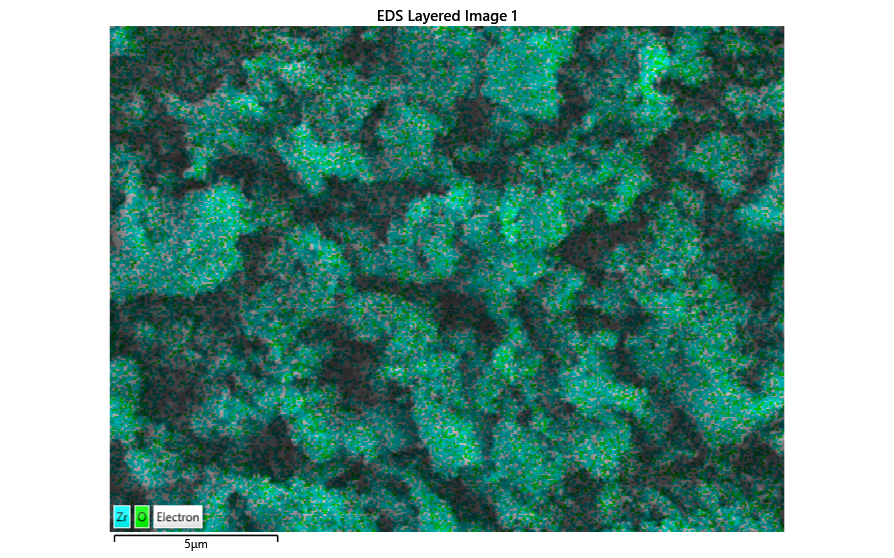


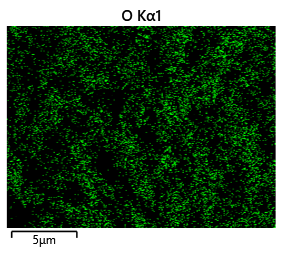


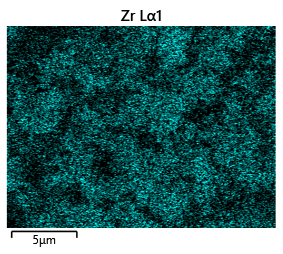


**Fig. S1:** EDS elemental mapping of nano-ZrO₂

Fig. S1. reveal an elemental mapping of nano-ZrO₂ showing the spatial distribution of zirconium (Zr) and oxygen (O). The EDS elemental maps confirm a homogeneous distribution of both elements over the entire analyzed area, indicating the formation of a chemically uniform ZrO₂ nanostructure. Quantitative EDS analysis reveals that zirconium and oxygen are present with weight percentages of approximately 74.4 wt% (Zr) and 25.6 wt% (O), respectively, which are in good agreement with the expected stoichiometry of ZrO₂.











**Fig. S2:** FESEM images of nano-ZrO₂ particles with different maginfication 5kx, 15kx, 30kx and 60kx.

FESEM images of nano-zirconium oxide (nano-ZrO₂) particles recorded at different magnifications (5k×, 15k×, 30k×, and 60k×). The nano-ZrO₂ particles are predominantly spherical and exhibit a highly uniform shape and spatial distribution, with an average particle size of below 50 nm. High-magnification FESEM micrographs (45,000× and 80,000×) further confirm this uniformity and reveal the absence of severe agglomeration, indicating good dispersion of the nanoparticles.











Fig. S3: Fig. S3. FESEM images of nano-polyether ether ketone (nano-PEEK) particles recorded at different magnifications (5k×, 15k×, 30k×, and 60k×). The particles exhibit a cubic-like morphology, along with the presence of smaller nanoparticles deposited on the surfaces of the larger cubic particles.

Fig. S3. FESEM images of nano-PEEK particles recorded at different magnifications (5k×, 15k×, 30k×, and 60k×). The nano-PEEK particles exhibit a wide size distribution ranging from approximately 26 to 100 nm and display a distinct cubic-like morphology. High-magnification FESEM micrographs (45,000×, 60,000×, and 80,000×; Fig. 1a) reveal well-defined angular particle boundaries and faceted surfaces characteristic of crystalline structures. In addition, smaller spherical-like nanoparticles are observed to be deposited on the surfaces of the larger cubic-like PEEK particles, forming a hierarchical nanostructure. This surface decoration with finer nanoparticles contributes to increased surface roughness and confirms the heterogeneous nanoscale morphology of the nano-PEEK powder.
